# Three-dimensional evaluation of hyoid bone position in nasal and mouth breathing subjects with skeletal Class I, and Class II

**DOI:** 10.1186/s12903-022-02257-4

**Published:** 2022-06-09

**Authors:** Amin S. Mohamed, Janvier Habumugisha, Bo Cheng, Minyue Zhao, Yucheng Guo, Rui Zou, Fei Wang

**Affiliations:** 1 Key Laboratory of Shaanxi Province for Craniofacial Precision Medicine Research, Clinical Research Center of Shaanxi Province for Dental and Maxillofacial Diseases, College of Stomatology, Xi’an Jiaotong University, Xi’an, 710004 Shaanxi People’s Republic of China; 2 Department of Orthodontics, Xi’an Jiaotong University, Xi’an, People’s Republic of China

**Keywords:** Hyoid bone, CBCT, Mouth breathing, Nasal breathing

## Abstract

**Background:**

This retrospective study investigated the effect of breathing pattern, skeletal class (Class I, Class II), and age on the hyoid bone position (HBP) in normodivergent subjects.

**Methods:**

A total of 126 subjects (61 males, 65 females) aged 7–9 years and 10–12 years were scanned using cone-beam computed tomography (CBCT). All participants were classified according to the anteroposterior skeletal pattern into (Class I, Class II). Each skeletal group was further divided according to the breathing mode into mouth breathers (MB) and nasal breathers (NB). The HBP was measured accordingly. Independent sample t-test and Mann Whitney U test were used to detect significant differences between the groups, and binary logistic regression was used to identify MB predictive indicators.

**Results:**

The breathing mode and skeletal class affected the vertical HBP in subjects with 7–9 years, while they affected the anteroposterior HBP in subjects with 10–12 years. Regarding the age effect, hyoid bone was located more anteriorly in the older NB subjects, and hyoid bone was more inferiorly in the older age group. A regression equation of the significant variables was formulated, C3-Me (P: 001, OR: 2.27), and H-EB (P: 0.046, OR: 1.16) were positively correlated with occurrence of MB.

**Conclusion:**

There were significantly different HBPs among subjects with different anteroposterior skeletal classes, breathing modes, and age cohorts. Moreover, C3-Me, and H-EB were significant predictors and correlated with increased likelihood of being MB subject.

## Background

The respiratory function has been linked to craniofacial development and occlusion. Poor nasal respiratory function is related to low airway capacity, which might lead to mouth breathing [1].

Multiple etiologies have been associated with mouth breathing: adenoid hypertrophy is considered the most common cause of mouth breathing in children [2]; other etiologies include tonsillar hypertrophy, nasal septum deviation, hypertrophied turbinate, and allergic rhinitis [3]. According functional matrix theory, in which he stated that development of the Moss who proposed nasal resistance caused by adenoid or skeletal tissues is guided by the soft tissues, therefore [4] of the craniofacial structures tonsillar hypertrohy are thought to affect the development.

Mouth breathing was considered a predisposing factor of obstructive sleep apnea syndrome [5]. A previous study [6] posited that a brachyfacial vertical skeletal pattern with a lower position of hyoid bone was a characteristic feature of OSAS patients.

Hyoid bone was described as a floating bone since it has no articulation with any other bone. It is linked to the pharynx, cranium, and mandible by muscles and ligaments, forming the oropharyngeal complex. It has three primary functions: deglutition, phonation, and breathing [7].

Many factors are related to HBP, changes in the position of the mandible whether skeletally, surgically, or after orthodontic treatment might result in a change in HBP and pharyngeal airway volume [8–11]. Because of the tight relationship between hyoid bone and pharyngeal airway, a better knowledge of the impact of the breathing pattern, skeletal class, and age on HBP would improve orthodontic diagnosis and treatment planning.

CBCT offered more precise information than lateral cephalogram (LC) [12]. Most previous studies used LC to determine HBP, and few of them used CBCT [13–18]. However, the effect of breathing mode on HBP using CBCT has never been studied previously. Hence, this study evaluated HBP in normodivergent children aged 7–12 years old with skeletal Class I and Class II and different breathing modes.

## Methods

### Ethical approval

This retrospective study was approved by the Ethics Review Committee of Xi'an Jiaotong University with protocol number Xjkqll [2018] No.17.

### Sample size calculation

Sample size was calculated using a formula proposed by Pandis [19] with power = 80%, level of significance = 0.05, to detect a difference of 4.44 mm in H–V distance between MB and NB and standard deviation = 4.2 mm [13]. We found that 14 subjects on each subgroup would be sufficient.

### CBCT process

Each patient was requested to sit straight and maintain maximal intercuspation of their jaws; their lips and tongue were assured to be in a resting condition. The Frankfort horizontal plane of the patients was maintained parallel to the ground, and patients were advised to breathe adequately via their nose, without swallowing or moving their head or tongue. All pictures were taken using (i-Cat, Imaging Sciences International, Hatfield, PA, USA) cone beam machine at 120 kV, 5 mA, 14 × 17 cm FOV, 0.4 mm voxel, and scan time of 8.9 s. After that, the CBCT pictures were stored as DICOM (digital imaging and communications in medicine) files.

### Subjects

**Fig. 1:**
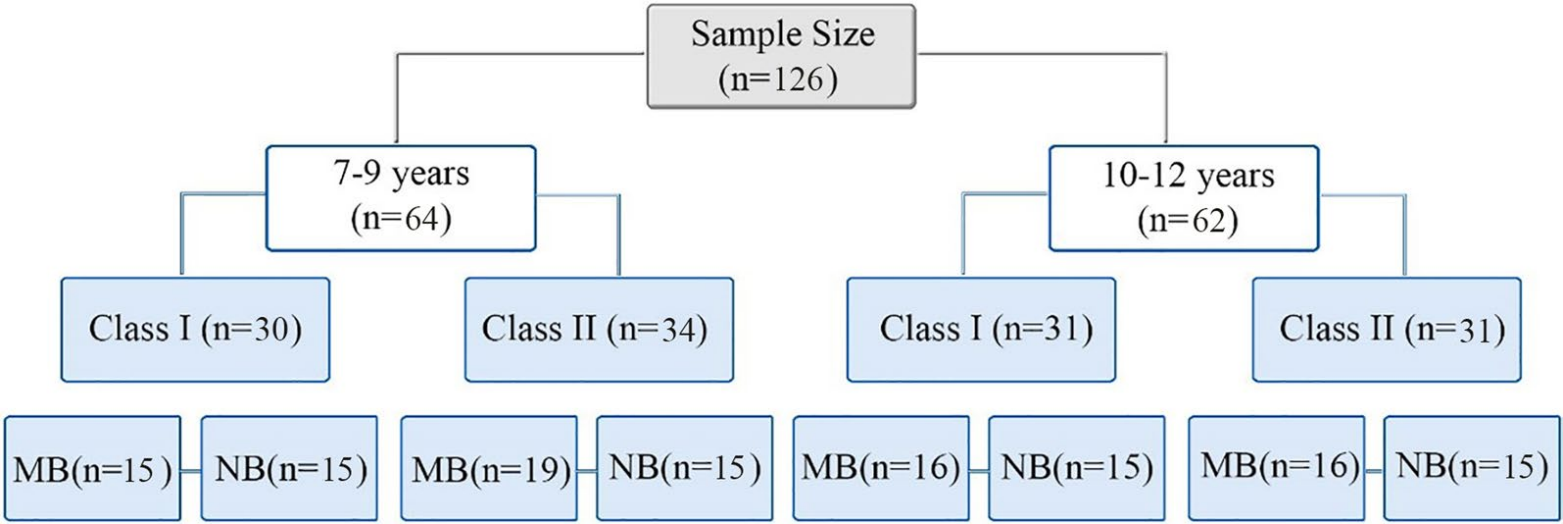
Sample distribution and grouping

In this study, 126 CBCT scans were collected from 61 males and 65 females. Eligible CBCT scans were obtained as a diagnostic record of children aged 7–12 years who first attended the Department of Orthodontics at Xi'an Jiaotong University from 2017 to 2021. Furthermore, prior to CBCT scanning, all patients' parents provided informed and written consent. CBCT scans with non-obvious hyoid bone and landmarks were excluded from the study, regarding the medical history, patients who had previously undergone orthodontic treatment, had enlarged tongue, or any syndromes in the head and neck area were also excluded from the study. The sample distribution of this study is illustrated in Fig. 1.

To identify the skeletal classes: ANB° from the cephalometric analysis of Steiner was used. Class I patients (1 ≤ ANB° ≤ 4.9), Class II patients (ANB° ≥ 5). All the patients were normodivergent growers (27 < FMA° < 37) [20], with (18.5 < BMI < 24) [8].

### Breathing pattern diagnostic criteria

Respiratory function was assessed by a multidisciplinary approach consisting of an expert orthodontist and an otolaryngologist. The orthodontist initially took a history from the children's parents about their children's sleeping habits, such as sleeping with their mouth open, drooling in pillow, and snoring, then performed a clinical examination of the children's habitual lip posture, nostril size and shape, and the Glatzel mirror test to identify mouth breathers [21].

Furthermore, all subjects were checked by an otolaryngologist, who confirmed the mouth breathers. A full examination by an otolaryngologist comprised a nasopharyngeal x-ray, rhinoscopy, and flexible nasopharyngoscopy. Mouth breathers were diagnosed based on the existence of nasopharyngeal airway obstruction caused by adenoid or tonsillar hypertrophy [22].

### CBCT orientation and measurements

**Fig. 2:**
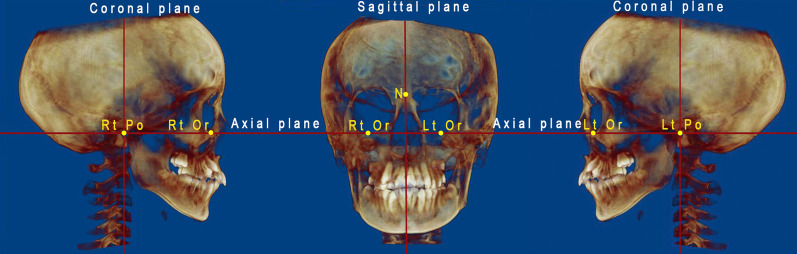
Adjustment of orientation planes

The DICOM files were imported into Dolphin Imaging software (version 11.7) (Dolphin Imaging & Management Solutions®, Chatsworth, CA, USA). All measurements were taken by a single investigator who was unaware of the participants' demographics. CBCT images were oriented, the axial plane was aligned with the Frankfort plane (FHP), and the midsagittal plane was aligned with the patient's midline, which is described as a vertical plane running across Nasion point (N), and the coronal plane was adjusted to be perpendicular to the axial plane and passing through Porion point (Fig. 2).

**Fig. 3:**
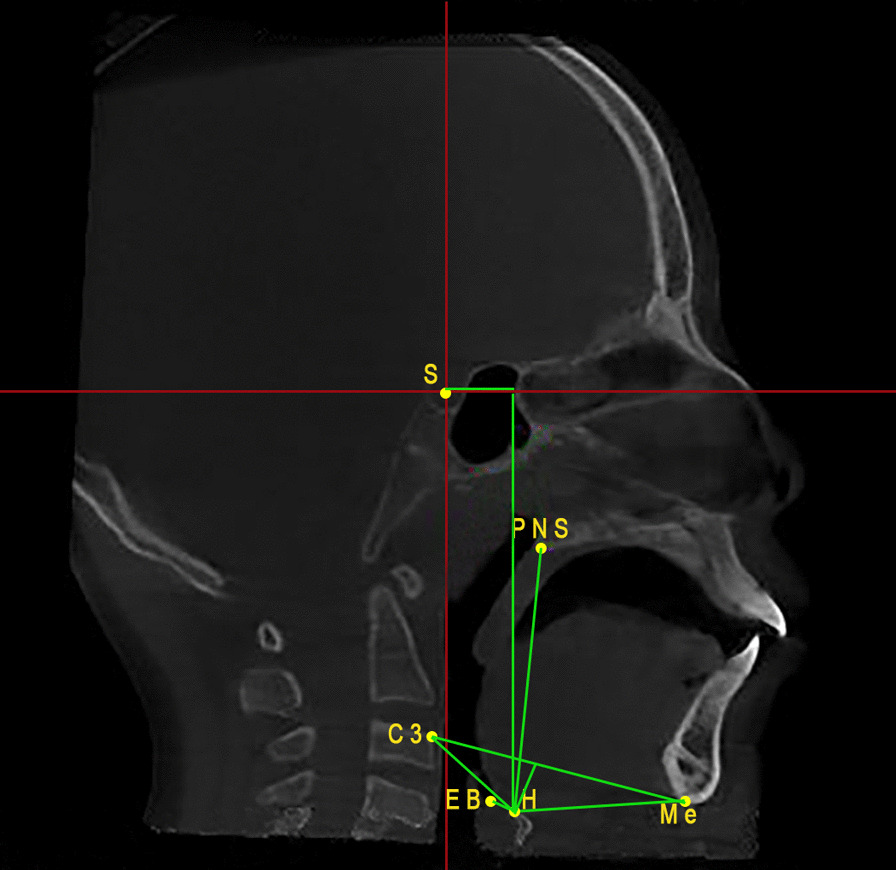
Hyoid bone landmarks and measurements

Hyoid bone measurements include eight linear measurements: H-Me, H-EB, C3-H, C3-Me, H-C3-Me, H-PNS, H–H, and H-V [14] (Table 1), (Fig. 3).

**Table 1:** Explanation of the landmarks and measurements

Measurement	Definition	Interpretation
H-Me	The distance between hyoid bone and Menton point	Hyoid bone position in relation to the mandible
H-EB	The distance between hyoid boneand Epiglottis point	Hyoid bone position in relation to the epiglottis
C3-H	The distance between the 3rd cervical vertebrae and hyoid bone	Hyoid bone position in relation to the 3rd cervical vertebrae
C3-Me	The distance between the 3rd cervical vertebrae and Menton point	This line forms the hyoid bone triangle combined with H-Me line, and C3-H line
H-C3Me	The perpendicular line from hyoid bone to the line connecting the 3rd cervical vertebrae and Menton point	The vertical height of the hyoid bone triangle. A positive value indicated a downward direction of the hyoid triangle, and a negative value indicated an upward direction of the hyoid triangle
H-PNS	The distance between hyoid bone and PNS point	Hyoid bone position in relation to the maxilla
H–H	The perpendicular distance running from hyoid bone to a vertical line extending from the Sella point	Horizontal position of hyoid bone
H-V	The perpendicular distance running from hyoid bone to a horizontal line extending from the Sella point	Vertical position of hyoid bone

H, the most superior point of hyoid bone; Me, Menton; EB, epiglottis base; C3, the most supero-anterior point of the 3rd cervical vertebrae; PNS, posterior nasal spine; S, Sella

### Statistical analysis

All measurements were analyzed with SPSS software (version 25.0, Chicago, Ill).

Shapiro–Wilk test was used to check the normality of variables. Among the total CBCT images, 30 images were randomly selected; Two investigators repeated all the measurements two weeks after the first measurement. The intra-investigator and inter-investigator errors were assessed by intraclass correlation coefficient. And Dahlberg’s formula $(\sqrt{\sum \frac{{D}^{2}}{2N}}$) was used to calculate the measurements error [23].

Independent sample t-test and Mann–Whitney U test were used for multiple comparisons between the groups, and logistic regression was used to identify MB predictive indicators; a *P* < 0.05 was considered significant.

## Results

The concordance index was ranged between 0.92 and 0.99 for the intra-investigator, and from 0.86 to 0.96 for the inter-investigator in the ICC test revealing high intra and inter-examiner reliability. Furthermore, the measurements errors ranged between 0.73 and 1.2 mm, so the errors were considered negligible.

After performing the Shapiro–Wilk test, some variables revealed non-normal distribution, so Mann Whitney U test was used for the non-normally distributed variables (H-EB, C3-H, C3-Me, H–H, H-V), and Independent sample t-test was performed for the normally distributed variables (H-Me, H-C3Me, H-PNS) to identify differences between the groups.

In the current study, the groups showed no significant difference in the demographic variables (Table 2).

**Table 2:** Descriptive statistics according to the demographics of age, anteroposterior skeletal class, and breathing mode

	7–9 years (n = 64)						10–12 years (n = 62)					
	Class I			Class II			Class I			Class II		
	Mouth breathing (n = 15)	Nasal breathing (n = 15)		Mouth breathing (n = 19)	Nasal breathing (n = 15)		Mouth breathing (n = 16)	Nasal breathing (n = 15)		Mouth breathing (n = 16)	Nasal breathing (n = 15)	
SNA°	79.44 (3.89)	79.27 (2.78)	NS	80.16 (3.35)	80.37 (3.53)	NS	80.76 (4.38)	80.84 (4.46)	NS	82.42 (3.46)	82.81 (2.68)	NS
SNB°	76.29 (2.98)	76.47 (2.20)	NS	73.69 (3.51)	74.00 (3.78)	NS	77.10 (4.19)	78.20 (4.20)	NS	71.07 (17.19)	76.36 (2.98)	NS
ANB°	3.52 (1.15)	2.83 (0.96)	NS	6.63 (0.81)	6.33 (0.53)	NS	3.64 (1.23)	2.87 (0.91)	NS	7.16 (1.17)	6.52 (0.76)	NS
FMA°	30.92 (3.78)	29.26 (1.87)	NS	31.71 (2.54)	30.10 (3.12)	NS	30.94 (3.56)	29.52 (2.58)	NS	31.31 (2.98)	30.01 (2.50)	NS
Age (years)	8.06 (0.88)	7.86 (0.83)	NS	8.15 (0.76)	8.20 (0.86)	NS	11.18 (0.75)	11.46 (0.83)	NS	10.68 (0.87)	11.26 (0.70)	NS

NS, not significant (*P* > 0.05) using Independent sample t-test

Class I and Class II patients with the same age group and breathing mode were compared. In 7–9 years group, there was significance related to the vertical parameters of hyoid bone between anteroposterior skeletal groups (H-C3Me, *P* = 0.0001; H-PNS, *P* = 0.0003; H-V, *P* = 0.002), indicating downward HBP in the Class I MB compared to class II MB. In contrast, Class I NB appeared with an upward HBP in relation to Class II NB group (H-V, *P* = 0.00007). For 10–12 years group significance was accompanied with the horizontal parameters of hyoid bone; class II patients had a backward HBP in both breathing patterns with weak significance for MB group (C3-H, *P* = 0.045 for MB, and C3H, *P* = 0.001 for NB) (Table 3).

**Table 3:** Comparison between Class I and Class II patients with similar age group and breathing mode

	7–9 years (n = 64)						10–12 years (n = 62)					
	Mouth breathing (n = 34)		*P* value	Nasal breathing (n = 30)		*P* value	Mouth breathing (n = 32)		*P* value	Nasal breathing (n = 30)		*P* value
	Class I (n = 15)	Class II (n = 19)		Class I (n = 15)	Class II (n = 15)		Class I (n = 16)	Class II (n = 16)		Class I (n = 15)	Class II (n = 15)	
	Mean (SD)	Mean (SD)		Mean (SD)	Mean (SD)		Mean (SD)	Mean (SD)		Mean (SD)	Mean (SD)	
H-Me^a^	39.82 (4.14)	39.56 (7.01)	0.901	41.56 (4.04)	38.33 (5.11)	0.065	42.60 (5.61)	40.30 (5.27)	0.241	42.72 (5.26)	41.09 (6.57)	0.46
H-EB^b^	8.45 (2.71)	9.82 (3.91)	0.339	7.21 (1.97)	6.76 (1.99)	0.506	10.08 (3.82)	7.88 (2.94)	0.054	11.88 (5.04)	8.66 (2.55)	0.077
C3-H^b^	25.12 (4.04)	25.42 (2.95)	0.741	24.87 (3.28)	25.68 (3.36)	0.663	27.61 (3.65)	25.10 (2.52)	0.045*	32.36 (5.06)	27.86 (2.88)	0.001**
C3-Me^b^	64.62 (4.40)	64.20 (7.55)	0.578	65.07 (5.38)	63.46 (6.63)	0.205	70.13 (7.61)	64.93 (6.68)	0.028*	71.90 (5.52)	65.14 (11.35)	0.029*
H-C3Me^a^	2.60 (1.88)	− 1.67 (3.54)	0.0001***	− 0.99 (4.36)	1.68 (3.17)	0.065	1.21 (4.83)	2.81 (4.16)	0.324	2.35 (7.62)	4.11 (4.18)	0.439
H-PNS^a^	51.04 (2.31)	45.80 ( 4.61)	0.0003***	46.82 (5.49)	49.54 (2.01)	0.082	51.97 (5.76)	53.92 (3.63)	0.261	51.92 (9.21)	53.65 (5.18)	0.532
H-H^b^	15.91 (17.72)	12.53 ( 8.66)	0.93	18.68 (9.60)	17.08 (5.99)	0.262	9.39 (6.58)	12.74 (8.85)	0.067	15.27 (8.47)	14.32 (5.34)	0.067
H-V^b^	82.38 (4.41)	77.37 (6.83)	0.002**	75.94 (2.99)	81.7 (3.50)	0.00007****	88.45 (6.86)	90.96 (6.21)	0.18	90.18 (11.34)	88.24 (6.92)	0.983

****p < 0.0001, ***p < 0.001, **p < 0.01, *p < 0.05,

^a^Independent sample t-test

^b^Mann–Whitney U test

In 7–9 years group, Class I MB presented with a downward HBP compared to their matched NB participants (H-C3Me, *P* = 0.006; H-PNS, *P* = 0.010; H-V, *P* = 0.0001). While class II MB patients were characterized by an upward HBP compared to their NB counterparts (H-C3Me, *P* = 0.007; H-PNS, *P* = 0.006; H-V, *P* = 0.007) (Table 4).

**Table 4:** comparison between mouth breathing and nasal breathing with matched age, and anteroposterior skeletal class

	7–9 years (n = 64)						10–12 years (n = 62)					
	Class I (n = 30)		*P* value	Class II (n = 34)		*P* value	Class I (n = 31)		*P* value	Class II (n = 31)		*P* value
	Mouth breathing (n = 15)	Nasal breathing (n = 15)		Mouth breathing (n = 19)	Nasal breathing (n = 15)		Mouth breathing (n = 16)	Nasal breathing (n = 15)		Mouth breathing (n = 16)	Nasal breathing (n = 15)	
	Mean (SD)	Mean (SD)		Mean (SD)	Mean (SD)		Mean (SD)	Mean (SD)		Mean (SD)	Mean (SD)	
H-Me^a^	39.82 (4.14)	41.56 (4.04)	0.254	39.56 (7.01)	38.33 (5.11)	0.573	42.6 (5.61)	42.72 (5.26)	0.951	40.3 (5.27)	41.09 (6.57)	0.712
H-EB^b^	8.45 (2.71)	7.21(1.97)	0.092	9.82 (9.82)	6.76 (1.99)	0.015*	10.08 (3.82)	11.88 (5.04)	0.285	7.88 (2.94)	8.66 (2.55)	0.363
C3-H^b^	25.12 (4.04)	24.87 (3.28)	0.884	25.42 (2.95)	25.68 (3.36)	0.754	27.61 (3.65)	32.36 (5.06)	0.002**	25.10 (2.52)	27.86 (2.88)	0.011*
C3-Me^b^	64.62 (4.40)	65.07 (5.38)	0.9	64.20 (7.55)	63.46 (6.63)	0.615	70.13 (7.61)	71.90 (5.52)	0.635	64.93 (6.68)	65.14 (11.35)	0.501
H-C3Me^a^	2.60 (1.88)	− 0.99 (4.36)	0.006**	− 1.67 (3.54)	1.68 (3.17)	0.007**	1.21 (4.83)	2.35 (4.83)	0.622	2.81 (4.16)	4.11 (4.16)	0.395
H-PNS^a^	51.04(2.31)	46.82 (5.49)	0.010*	45.8 (8.66)	49.54 (2.01)	0.006**	51.97 (5.76)	51.92 (9.21)	0.986	53.92 (3.63)	53.65 (5.18)	0.866
H-H^b^	15.91 (17.72)	18.68 (9.60)	0.17	12.53 (8.66)	17.08 (5.99)	0.076	8.88 (5.71)	15.27 (8.47)	0.036*	12.74 (8.85)	14.32 (5.34)	0.968
H-V^b^	82.38 (4.41)	75.94 (2.99)	0.0001***	77.37 (6.83)	81.70 (3.50)	0.007**	88.45 (6.86)	90.18 (11.34)	0.874	90.96 (6.21)	88.24 (6.92)	0.205

****p < 0.0001, **p < 0.01, *p < 0.05

^a^Independent sample t-test

^b^Mann–Whitney U test

For the 10–12 years group, Class I MB compared to the control NB group displayed a backward HBP in relation to S point and the 3rd cervical vertebrae (C3-H, *P* = 0.002; H–H, *P* = 0.036). Class II MB group showed a backward HBP in relation to the 3rd cervical vertebrae (C3-H, *P* = 0.011). No significant difference between MB and NB was detected regarding the vertical HBP for this age group (Table 4).

On the other hand, the effect of age on the HBP was considerably detected. After controlling the effect of the antero-posterior skeletal Class and breathing mode, hyoid bone vertical distance (H-V) was significantly increased in 10–12 years group than 7–9 years group in all skeletal classes and breathing mode categories (Table 5).

**Table 5:** Effect of age on HBPs in each skeletal class and breathing pattern

	Class I (n = 61)						Class II (n = 65)					
	Mouth breathing (n = 31)		*P* value	Nasal breathing (n = 30)		*P* value	Mouth breathing (n = 35)		*P* value	Nasal breathing (n = 30)		*P* value
	7–9 years (n = 15)	10–12 years (n = 16)		7–9 years (n = 15)	10–12 years (n = 15)		7–9 years (n = 19)	10–12 years (n = 16)		7–9 years (n = 15)	10–12 years (n = 15)	
	Mean (SD)	Mean (SD)		Mean (SD)	Mean (SD)		Mean (SD)	Mean (SD)		Mean (SD)	Mean (SD)	
H-Me^a^	39.82 (4.14)	42.60 (5.61)	0.129	41.56 (4.04)	42.72 (5.26)	0.503	39.56 (7.01)	40.3 (5.27)	0.731	38.33 (5.11)	41.09 (6.57)	0.209
H-EB^b^	8.45 (2.71)	10.08 (3.82)	0.154	7.21 (1.97)	11.88 (5.04)	0.007**	9.82 (3.91)	7.88 (2.94)	0.119	6.76 (1.99)	8.66 (2.55)	0.026*
C3-H^b^	25.12 (4.04)	27.61 (3.65)	0.068	24.87 (3.28)	32.36 (5.06)	0.00008****	25.42 (2.95)	25.10 (2.52)	0.973	25.68 (3.36)	27.86 (2.88)	0.081
C3-Me^b^	64.62 (4.40)	70.13 (7.61)	0.017*	65.07 (5.38)	71.90 (5.52)	0.003**	64.20 (7.55)	64.93 (6.68)	0.596	63.46 (6.63)	65.14 (11.35)	0.146
H-C3Me^a^	2.60 (1.88)	1.21 (4.83)	0.307	− 0.99 (4.36)	2.35 (7.62)	0.151	− 1.67 (3.54)	2.81 (4.16)	0.001***	1.68 (3.17)	4.11 (4.18)	0.083
H-PNS^a^	51.04 (2.31)	51.97 (5.76)	0.566	46.82 (5.49)	51.92 (9.21)	0.076	45.80 (4.61)	53.92 (3.63)	0.0001****	49.54 (2.01)	53.65 (5.18)	0.007**
H-H^b^	15.91 (17.72)	8.88 (5.71)	0.322	18.68 (9.60)	15.27 (8.47)	0.28	12.53 (8.66)	12.74 (8.85)	0.753	17.08 (5.99)	14.32 (5.34)	0.319
H-V^b^	82.38 (4.41)	88.45 (6.86)	0.026*	75.94 (2.99)	90.18 (11.34)	0.00002****	77.37 (6.83)	90.96 (6.21)	0.00003****	81.70 (3.50)	88.24 (6.92)	0.007**

****p < 0.0001, ***p < 0.001, **p < 0.01, *p < 0.05

^a^Independent sample t-test

^b^Mann-Whitney U test

Additionally, Class I MB group showed significantly increased C3-Me distance in 10–12 years group compared to 7–9 years group (*P* = 0.017). While Class I NB group had significantly increased H-EB, C3-H, and C3-Me distances (*P* = 0.007, 0.00008, 0.003), respectively. Class II MB showed significantly increased differences in 10–12 years group in H-C3Me (*P* = 0.001) and H-PNS (*P* = 0.0001). While Class II NB showed significance in H-EB, and H-PNS (*P* = 0.026, 0.007) (Table 5).

The regression equation was formulated as Y = 0.11 − 0.769 * X1 + 0.151 * X2 − 0.969 * X3 + 0.823 * X4 − 0.064 * X7 (Y: Breathing pattern Nasal breathing or Mouth breathing; X1: H-Me; X2: H-EB; X3: C3-H; X4: C3-Me; X7: H–H). According to the equation, C3-H was the most influencing factor, followed by C3-Me, H-Me, H–H, and H-EB (*P* value: 0.0001, 0.001, 0.001, 0.046, and 0.020 respectively); considering that the negative β value indicated decreased likelihood of falling into MB group, while the positive β value indicated increased likelihood of falling into MB group. (Table 6).

**Table 6:** Binary logistic regression analysis

	β	SE	*P* value	Odds ratio	95% CI for odds ratio	
					Lower	Upper
H-Me	− 0.769	0.24	0.001**	0.463	0.287	0.734
H-EB	0.151	0.076	0.046*	1.163	1.003	1.343
C3-H	− 0.969	0.245	0.0001***	0.379	0.233	0.607
C3-Me	0.823	0.244	0.001**	2.278	1.42	3.697
H-C3Me	0.016	0.088	0.851	1.017	0.863	1.207
H-PNS	0.054	0.082	0.509	1.056	0.898	1.238
H–H	− 0.064	0.028	0.020*	0.938	0.888	0.989
H-V	− 0.01	0.054	0.849	0.99	0.895	1.078
Skeletal class	0.023	0.428	0.958	1.023	0.192	2.325
Age group	0.147	0.578	0.8	1.158	0.385	3.597
Constant	0.11	5.324	0.983	1.117		

***p < 0.001, **p < 0.01, *p < 0.05

## Discussion

Our findings asserted that there were differences in HBP between MB and NB children, in addition antero-posterior skeletal Class (Class I and Class II), and age affected HBP.

Different methodologies had been used to evaluate the HBP; Lateral cephalometric was more predominant for determination of HBP [5, 24, 25]. As reported previously, the CBCT scan obtained before orthodontic diagnosis and treatment planning can help in gaining a clear clinical judgement of hyoid bone position and its surrounding structures [14]. Numerous CBCT studies [17, 18] had evaluated HBP in nasal breathing subjects. The current study used CBCT to evaluate the effect of different breathing patterns on HBP.

All CBCT images were taken when only CBCT was expected to add additional information which would aid in orthodontic diagnosis and treatment planning. The authors’ institution follows the ALARA principle “as low as reasonably achievable” [26] ensuring not to expose the patients to unnecessary ionizing radiation.

We believe that recognition of mouth breathing patients should be conducted through a multidisciplinary approach by orthodontists and otolaryngologists as recommended by Costa et al. [3] Previous literature relied on visual and clinical examination only to diagnose mouth breathing, which in fact could lead to improper mouth breathing recognition protocol [27]. This might clarify the different findings and contradictions between various researchers.

Few studies relied on otolaryngologists' diagnosis of mouth breathing [22, 28]. However, previous literature found that orthodontists can accurately diagnose nasal breathing and advised collaboration between orthodontists and otolaryngologists regarding mouth breathing diagnosis [3]. Lymphoid tissue develops fast after birth, reaches its peak size in childhood, begins to regress around the age of 8–10 years, and usually entirely diminishes around 12–14 years [29]. Hence, in our study: the history taking and clinical tests were used as preliminary screening tests for detection of mouth breathing, and the otolaryngologists confirmed the diagnosis, cooperation between the two disciplines led to a better diagnosis and treatment planning [3].

It has been documented that the HBP could be influenced by the anteroposterior sagittal skeletal patterns [9], vertical skeletal patterns [30], and age [31]. In our study, patients with similar age cohorts and skeletal patterns were compared to detect the differences in HBP between MB and NB, given that all of our participants were normal vertical growers.

Chung et al. [32] compared mouth breathing and nasal breathing children, concluded that mouth breathing patients had elevated HBP compared to the nasal breathing children, although they didn't classify their participants into different antero-posterior sagittal classes, but they found that most of mouth breathing participants had a tendency toward having Class II malocclusion, this concurred with our finding that Class II MB children aged 7–9 years exhibited an upward HBP compared to Class II NB with similar age group. Furthermore, this finding was also corroborated by Chaves et al. [33] who emphasized that asthmatic patients with mouth breathing had an elevated HBP in relation to the mandible and 3^rd^ cervical vertebrae.

However, Cuccia et al. [24] and Behlfelt et al. [34] claimed that mouth breathing children showed extended head posture as well as a lower HBP. Our study found that in 7–9 years group, MB children with skeletal Class I displayed a lower HBP than their matched NB group. A recent study by Vuong and Kang [16] found a positive correlation between the superior HBP and the constricted airway. Furthermore, skeletal class II pattern and mouth breathing habit have been stated to predispose to a constricted airway [22, 35]. This could explain our finding that MB patients with Class II pattern exhibited more superior HBP than Class I MB patients.

Behlfelt et al. [34] suspected that the lower HBP in mouth breathers was attributed to lower position of the tongue and to allow for more airway patency as the airway volume might be decreased in the mouth breathers [29], whereas, Chaves et al. [33] suggested that the upward HBP is a compensatory mechanism; as mouth breathing is accompanied with clockwise rotation of the mandible, which might release the tension applied by suprahyoid muscle to hyoid bone, thus led to an inferior HBP and constricted pharynx as well, then mouth breathers tended to extend their heads to allow for more airway patency, this posture exaggerated the tension applied by suprahyoid muscle, which consequently pulled hyoid bone to a superior position. Moreover, in our study, we also used Sella point to determine HBP as it is a more stable reference point than cervical vertebrae and mandible [32].

According to Janicka and Halczy-Kowalik [36] who assessed the different HBP in mouth and nasal breathers, their sample ranged between 9 and 35 years old; they presumed that mouth breathers had a backward HBP described by the parameter (C3-H). This finding was consistent with our findings in 10–12 years group, in which MB in both skeletal classes demonstrated a backward HBP. However, this finding is contradicted with other findings expressed by Juliano et al. [5], who assumed an anterior HBP for mouth breathers which is attributed to their head extension to enhance breathing capacity. However, it is worthy to note that previously mentioned studies used varied study designs and methodologies to evaluate HBP.

In the current study, hyoid bone descent in the older age group. Pae et al. [6] conducted a longitudinal study and described the early descent of hyoid bone as a physiological phenomenon related to speech development, while late descent is associated with increased resistance of airway with aging which usually seen on OSAS patients.

Mouth breathing habit was reported to cause alteration in the normal growth of the craniofacial complex and could be a risk factor for developing OSAS [5]. Our regression model was capable of predicting mouth breathers based on hyoid bone measurements with 76.2% overall accuracy. Moreover, C3-Me and H-EB were found to be significant predictors, for each one-unit increase in the previously mentioned predictors the odds of being mouth breather increase by 2.27 and 1.16, respectively. Increased values of these parameters speculate that MB subjects had extended head posture and hyoid bone modifies its position to enhance breathing capacity [34].

Previous studies have shown an association between HBP and the severity of OSAS. Chang and Shiao [37] reported that the distance from the hyoid bone to the mandibular plane (MP-H) was positively correlated to the Apnea Hypopnea Index (AHI), and subjects with longer MP-H distance suffered from more daytime sleepiness. A recent review by Haskell et al. [38] discussed the favorability of AHI response to oral appliances. Treatment favorability was linked to the anatomical position and angulation of hyoid bone beside maxillofacial and pharyngeal parameters. Moreover, several studies reported hyoid bone positional changes after orthodontic treatment with functional appliances [39, 40]. Such findings highlight the need of gaining a better knowledge of HBP to permit early intervention and a better prognosis.

Our study demonstrated in-depth grouping for age and anteroposterior skeletal classes to detect the differences in HBP between NB and MB, but one limitation is we didn't include Class III patients and we didn’t consider gender differences due to the lack of samples; another limitation is our cross-sectional retrospective study is not capable to assess the causality principle; the differences between NB and MB were detected on one-time point, However, this study adopted a cross-sectional design because of the ethical concerns associated with possibility of increased radiation doses accompanying longitudinal studies; also we didn't include subjects less than 7 years old or older than 12 years old, and this study only included Chinese participants.

## Conclusion

1. The current study found that individuals with different anteroposterior skeletal classes, breathing mode, and age showed significantly different HBPs.
2. C3- Me and H-EB were significant predictors and positively correlated with increased probability of impaired breathing function.

## Data Availability

Data used and/or analyzed during the current study are available from the corresponding author on reasonable request.

## Abbreviations

OSAS: Obstructive sleep apnea syndrome
LC: Lateral cephalogram
MB: Mouth breathing
NB: Nasal breathing
CBCT: Cone-beam computed tomography
HBP: Hyoid bone position
3D: Three dimensional
DICOM: Digital imaging and communication
ICC: Intra-class correlation coefficient
